# Visualization of superficial vein dynamics in dorsal hand by near-infrared imaging in response to elevated local temperature

**DOI:** 10.1117/1.JBO.26.2.026003

**Published:** 2021-02-23

**Authors:** Mohiuddin Khan Shourav, Jungil Choi, Jung Kyung Kim

**Affiliations:** a Kookmin University, Graduate School, Department of Mechanical Engineering, Seoul, Republic of Korea; b Kookmin University, School of Mechanical Engineering, Seoul, Republic of Korea; c Kookmin University, Graduate School, Department of Integrative Biomedical Science and Engineering, Seoul, Republic of Korea

**Keywords:** near-infrared imaging, vasomotor response, vein diameter, skin temperature, vein puncture, vein cannulation

## Abstract

**Significance:** Dry or moist skin-contact thermal stimulation for vein puncture (VP) and vein cannulation (VC) may not be feasible for sensitive skin. For a damaged, burned, or dark skin, near-infrared (NIR) imaging is preferred to visualize a vein. Postprocessing of NIR images is always required because the skin is a reflective material and veins need segmentation for quantitative analysis.

**Aim:** Our pilot study aims to observe the effect of noncontact local heating on the superficial metacarpal veins in the dorsal surface of the hand and to visualize vein dynamics using an NIR imaging system.

**Approach:** Our experiment consists of studies A and B at two ambient temperatures, 19°C and 25°C. A simple reflection-based NIR imaging system was installed to acquire sequential vein images for 5 min before and after applying 10 min of radiant thermal stimulation. To measure the vein diameter (VD), we trained a convolutional neural network (CNN) on sequential raw images to predict vein-segmentation masks as output images. Later these masked images were postprocessed for the VD measurements.

**Results:** The average VD was significantly increased after thermal stimulation in study A. The maximum increments in VD were 39.3% and 9.19%, 1 min after thermal stimulation in studies A and B, respectively. Both the VD and skin temperature ($T_{\text{skin}}$) follow negative exponentials in time, and the VD is proportional to $T_{\text{skin}}$. A multiple linear-regression model was made to predict the final VD. A significant difference was observed in the change of the VD.

**Conclusions:** NIR imaging with CNN can be used for quantitative analyses of vein dynamics. This finding can be further extended to develop real-time, image-guided medical devices by integrating them with a radiant heater and to assist medical practitioners in achieving high success rates for VP or VC.

## 1 Introduction

The superficial veins of the upper limb, especially the dorsal metacarpal veins, are always the first choice to access during vein puncture (VP) and vein cannulation (VC). Enhancing the techniques of performing VP or VC is very important. Procedural successes recorded correlate with features such as technical skill, experience, and the size of veins. It has been reported that factors such as obesity, dark skin, intravenous drug abuse, prior chemotherapy, and old age can increase the difficulty associated with VP or VC.^1^^,^^2^ The sympathetic nervous system is the most important vasopressor system in the control of venous capacitance.^3^ Venoconstriction is caused by numerous factors, including hypothermia, hypotension, caffeine or nicotine use, medications (e.g., noradrenaline, 5-hydroxytryptamine, and ergot derivatives), pain from repeated attempts at VP or VC, and fear of the procedure. These factors can make venous access more difficult.^1^^,^^4^ There has been a report that peripheral VC failure is 12% to 40% in adults and 24% to 64% in children.^5^

It is well known that the most common sites for venous access are the superficial veins of the upper limb, particularly dorsal metacarpal veins and the median cubital vein. Even though dorsal metacarpal veins are the first choice for venous cannulation, their anatomic variation information is scarce.^6^ Several techniques have been reported to improve the success of VP or VC of veins in adults and children.^7^^–^^9^ As of the time of this study, techniques employed to improve vein access in both standard and emergency situations include venous visualization techniques [transillumination through the use of normal light, infrared (IR), computed tomography, ultrasonography, or sonography] and vasodilation techniques (e.g., the use of gravity, fist-clenching, vein tap and milking, tourniquet application, the use of local vasodilators such as nitroglycerin, applying topical heat, and stimulating the surface veins by striking them). A dry heat increases vein diameter (VD) more than moist heat, as reported in many studies.^10^

However, most often, a skin-contact dry or moist heat is applied to the target area, but that may not always be feasible for sensitive skin. Also, in case of damaged, burned, or dark skin, it is difficult to observe the vein without an imaging technique. Postprocessing of near-infrared (NIR) images is always required as the skin is very reflective material and veins need to be segmented for quantitative analysis.

Difficulty in vein visualization is a common complication that leads to the repetition of VP or VC procedures.^10^ This repeated attempt could produce a recurrent vasoconstriction effect. Noncontact heating to the target area would be useful when the patient’s skin is sensitive to the touch of other material for heating (for example, rash or close to burn or burned skin that cannot be seen under normal light; NIR is applicable here). Therefore, we investigated the vasodilation induced by noncontact thermal stimulation on the hand’s dorsal part under an NIR imaging system. We observed that the increment of local temperature at the dorsal surface of the hand increases the VD, which may facilitate the VP and VD procedures. We also found that different ethnic groups exhibit different vein dynamics and skin temperatures ($T_{\text{skin}}$) in response to the same intensity of thermal stimulation. An empirical relation is proposed between the $T_{\text{skin}}$ and VD.

## 2 Materials and Methods

### 2.1 Study Design and Participants

We designed our study by comparing before and after noncontact thermal stimulation on the dorsal metacarpal veins using the two-dimensional (2D) VD measured using NIR imaging system. We recruited university students in the Republic of Korea, aged 20 to 32 years, and 10 volunteers were chosen to participate in the experiments; the demographic characteristics are shown in Table 1. We limited the participants to avoid the following set of people: (1) those receiving treatment for skin disease, (2) those with a wound or eczema at the target site, (3) those with peripheral vascular disease, diabetes, or peripheral neuropathy, (4) those with anticoagulant treatment and findings of infiltration and phlebitis, and (5) those with allergies or sensitivity to heat application. We limited the target site to the dorsal surface of the hand as this part of the body is mostly naked and easy to expose under thermal stimulation.

**Table 1:** Demographic characteristics of the participants.

Parameter	Study A ($T_{\text{room}}=19{}^{\circ}C$)	Study B ($T_{\text{room}}=25{}^{\circ}C$)
Age (years)	26±2.3	
BMI ($\mathrm{kg}/m^{2}$)	23.2±2.7	
Prewarming VD (mm)	2.29±0.45	3.08±0.64

Note: All data were presented mean ± SD (standard deviation). n=10; BMI = Body-mass index.

Participants were advised not to engage in any intense physical exercise for 12 h before the experimental session as it was reported that veins would be dilated due to body exercise.^11^

### 2.2 Target Site of Blood Vessel

Most of the blood vessels are identified through the image sequence. The average diameter of vein for an individual is considered for our analysis. Dorsal metacarpal blood vessels located on the dorsal surface of the hand were chosen for our experiment. As the VD varies person to person, the analysis was done by measuring the percentage of its maximum dilation. Arterioles, capillaries, and venules are different types of blood vessels responding differently to various stimuli.^12^^–^^14^ Vessel types can be classified by their diameters. The targeted sites are nondominant (mostly left hand) and peripheral dorsal surface of the hand. We define our target veins as those present in the target site and having a diameter ≥1.1  mm. It should be noted that if we found no suitable match of the vein, we excluded the data for subsequent analysis.

### 2.3 Environment and Thermal Stimulation

We performed our thermal stimulation experiments at two different room temperatures ($T_{\text{room}}$) of 19°C and 25°C for studies A and B, respectively, with an approximation of 50% relative humidity confirmed by a humidity sensor (DHT22, Adafruit, New York). The sensors have ±0.5°C and 2% to 5% accuracy for temperature and humidity measurements, respectively.

**Fig. 1:**
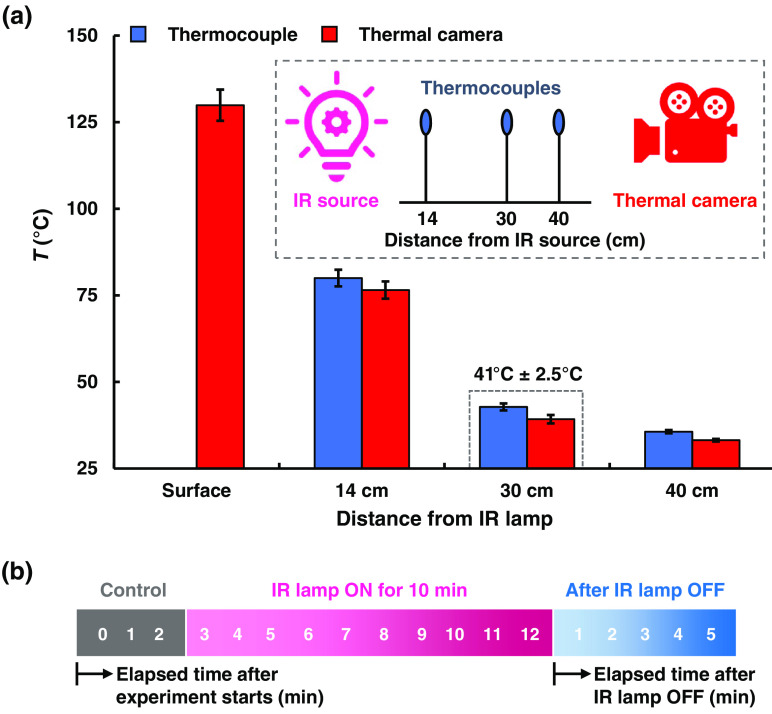
(a) Optimal position of the heat source to achieve a target temperature was determined by measuring the temperature at different distances from an IR radiant lamp. The inset shows a schematic for measurement of temperature using a thermocouple and a thermal camera. (b) Procedure for human subject experiment.

Each participant rested in the experiment room in a supine position for 10 min. After resting, the participant’s $T_{\text{skin}}$ was measured at the target site by attaching a skin-surface probe (SST-1, Physitemp Instruments, New Jersey), which has an accuracy of ±0.1°C on the skin. A near-infrared (NIR) imaging system was placed above the hand to capture sequential images before and after applying thermal stimulation. We irradiated the dorsal surface of the hand using a noncontact 250-W IR light (Infralux 300A, Daekyung Co. Ltd., Pocheon, Republic of Korea), with the bulb-surface temperature maintained at 129.9°C±4.5°C as measured with a thermal camera (A655sc, FLIR, Wilsonville, Oregon). Many studies have confirmed that local thermal stimulation for 15 min can increase the VD;^1^ however, most nurses apply thermal stimulation for less than 15 min.^15^ Because we designed our study with 10 min of thermal stimulation, we chose 42°C as our target temperature as several studies have reported that a vein can be dilated adequately at this temperature.^7^ We measured the temperature at different distances from the heat source to find an optimal distance, which was found to be 30 cm as shown in Fig. 1(a). The IR lamp was placed at the position to provide radiant thermal stimulation at the dorsal surface of the hand for 10 min to increase $T_{\text{skin}}$. The temperature produced by the radiation heat source was 41°C±2.5°C as confirmed with a thermocouple and a thermal camera. Moreover, this temperature is sufficiently low to prevent burning the skin as reported previously.^16^^,^^17^ Figure 1(b) shows the procedure for each human subject experiment. NIR images were recorded for 2 min before thermal stimulation and the vein image taken at 2 min was set as the control. After 10-min thermal stimulation on the hand surface, the vein images were recorded for 5 min at an interval of 1 min. All human imaging and measurements were performed in compliance with guidelines and regulations issued by the Institutional Review Board of Kookmin University.

### 2.4 Measurement of VD

**Fig. 2:**
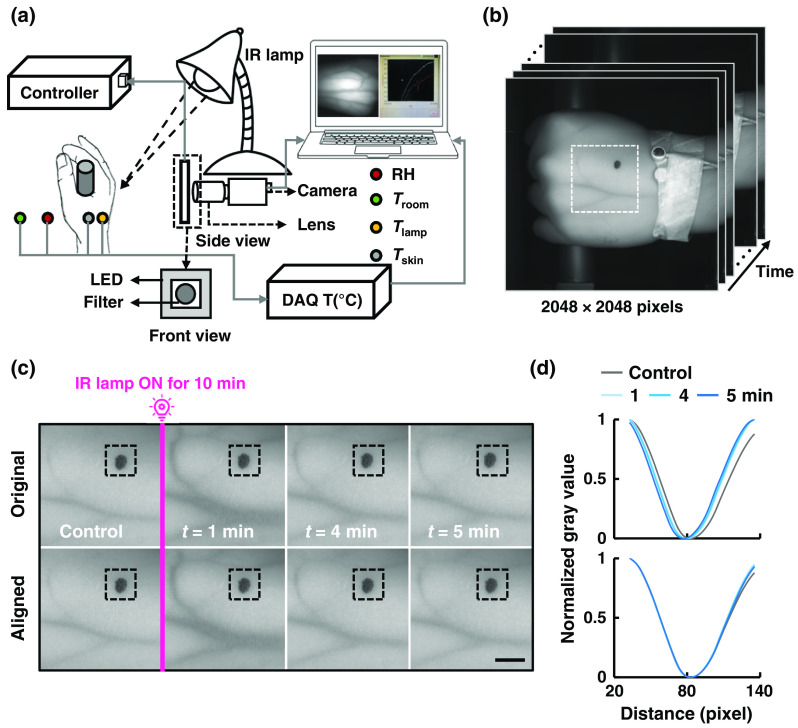
Acquisition and alignment of images at the experiment. (a) A schematic of the experimental setup for NIR image acquisition. (b) Image sequence stored in the memory for further analysis. (c) The inset in (b) as ROI is cropped and showed for geometrical correction. Upper row represents the original cropped raw images and lower row shows the geometrically corrected images. A square dashed box is drawn on the raw image by considering the dark mark at its center. Scale bar = 10 mm. (d) Quantitative analysis of the original cropped and corrected image are shown by a normalized gray value inside the dashed box centering the dark mark as shown in (c).

NIR rays penetrate skin and tissue deeper than the visible light. An NIR light source can facilitate imaging the vein through the skin tissue. About 95% of the dry weight of red blood cells in the blood is hemoglobin and the hemoglobin in the venous blood is deoxyhemoglobin.^18^ We considered the NIR imaging system assembled with several pieces of equipment, such as an NIR CCD camera (Grasshopper3 GS3-U3-41C6NIR-C, FLIR, Oregon) and a high-resolution lens (LYM1614, Tuss Vision Inc., Tokyo, Japan) with a bandpass filter (BP850, Midwest Optical Systems, Inc., Palatine, Illinois) in measuring VD. An NIR light-emitting diode (LV-ILA-94SF-IR-850, LVS Co. Ltd., Incheon, Republic of Korea) light source was used for illuminating the veins on the dorsal side of the hand. A schematic of the imaging setup is shown in Fig. 2(a).

To make a stable prediction of the VD in a certain time interval, geometrical correction of the input image is crucial. As we have recorded the NIR image sequence for 7 min with an interval of 1 min, the stabilization of the sequential images was necessary. The first frame of each recorded image sequence was imported. A region of interest (ROI) with a high-contrast edge was selected as shown in Fig. 2(c). The high-contrast edge inside the ROI made the stabilization less prone to errors. Motion vectors were estimated using the “block-matching algorithm.”^19^ “Exhaustive search/full search” was used in a predefined search region outside the ROI boundary. We calculated a translation offset from the calculated motion vector and translated the frame to the newly computed location from the translation offset with the same initial image size. The above steps were repeated for all frames in the whole sequence.

The raw image sequence as shown in Fig. 2(b) was stored in the memory for postprocessing by geometrical correction. This processing helped to line up the image sequence as it became aligned. To carry out our measurement, we made a mark on each participant’s hand during imaging as shown in Fig. 2(c). A quantitative analysis of the geometrical reconstruction can be explained by Fig. 2(d). A square ROI was drawn as a dashed line by considering the dark mark at its center. The actual gray value of the raw image was extracted from the ROI, which generated an inverted Gaussian-shaped curve. The intensity profile of the dashed box was measured using ImageJ. The measured intensity profile of the dashed box was normalized later and shown in Fig. 2(d). Randomly distributed Gaussian curves of the average gray value shown at the upper plot of Fig. 2(d) became more uniform after applying the geometric correction, as shown at the lower plot of Fig. 2(d). Furthermore, we trained the sequential raw images using convolutional neural network (CNN) to predict the vein-segmentation mask as output images. The CNN deep learning model used in this study was greatly inspired and based on previous study done by Ronneberger et al.^20^ This model architecture is based on an encoder–decoder network. Half of the network generates the feature space and the second half decodes the generated features to predict the target data. The input and output of the network is 256×256. After the input layer, there are several convolutions (3×3) and pooling layers (2×2) to generate the features. To decode the features to target, several back-to-back convolution and upsampling layers are used.

During the training, few images were masked manually to compare the output. The processed images were further put into a MATLAB^®^ (MathWorks, Natick, Massachusetts) algorithm platform to measure its diameter.

### 2.5 Quantitative Analysis of VD

**Fig. 3:**
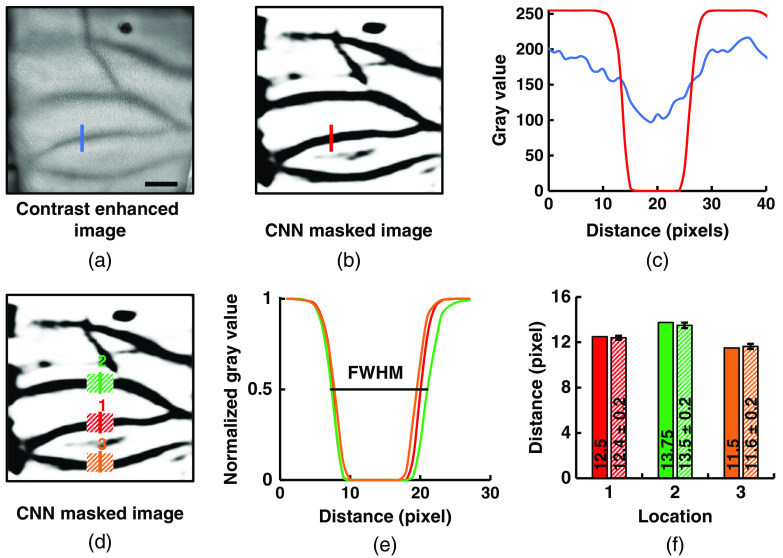
Quantitative measurement of VD. (a) Vein existing ROI is cropped from the raw image for processing. Scale bar = 10 mm. (b) Raw image was further processed for VD measurement. (c) A quantitative measurement of an arbitrary vein is scanned in 1D position to emphasize the reason for raw image processing. The blue and red lines in the plot represent gray values of the raw and processed images, respectively. Red color line shows a significant difference from the blue line, which are used to clearly determine VD. (d) However, 1D scan gray value measurement could be confusing as the vein is not symmetric in size for its whole length. A random area from the target vein is selected to measure its average. (e) The dark red, green, and orange lines in the plot are the 1D scan gray values, respectively, for 1, 2, and 3, as shown in (d). The VD is measured by determining FWHM of the Gaussian curve. (f) A quantitative analysis is shown in the dark and shaded bar plot for VD measurement in single line scan and area scan, respectively.

An ROI was cropped by considering vein existence from the raw image. The difference of the gray value between the cropped raw image and the CNN-masked image was very easy to observe as shown in Fig. 3(c). We performed a one-dimensional (1D) scan to measure VD. The observed VD was measured from Figs. 3(a) and 3(b) by drawing a single line over a vein. However, we found that there was a slight difference of pixel values along the vein. So, we selected a certain region of the vein as the dashed box in Fig. 3(d) to measure its average diameter as shown in Fig. 3(f). The regions were selected manually with the following criteria.

The prominent vein sites, which were clearly visible across all the images, were selected. It ensured justified measurement in the full image sequence. As the prominent vein sites give better visibility to NIR imaging, the change of diameter at that site would also be visible easily. For this reason, these selections may produce a higher degree of correlation upon further analysis. To determine the VDs from the selected regions, the prominent vein sites were chosen by drawing polylines inside the vein regions. The drawn lines were used as guides to indicate the vein regions in the algorithm. The algorithm then sliced the NIR image in the direction of the drawn lines and the diameter was calculated by peak detection of the inverted image from each slice. The full width at half maximum (FWHM) of the peaks detected were considered as the VD of each slice in pixel unit. The calculated VD was averaged and presented as the average diameter of each region selected with an individual polyline.

VD for 1D selection was determined by measuring the FWHM, as shown in Fig. 3(e). We observed a clear difference between single line and area scan observations. The average of VDs from the scan area was used for analysis in the rest of this study. One representative participant’s VD is displayed with a unit of pixel in Fig. 3 as an example.

### 2.6 Measurement of Vital Signs

We measured tympanic temperature ($T_{\text{tym}}$), heart rate (HR), and systolic blood pressure (${\mathrm{BP}}_{\text{sys}}$) of an individual before and after thermal stimulation. $T_{\text{tym}}$, HR, and ${\mathrm{BP}}_{\text{sys}}$ were measured by IR ear thermometer (PHTM20BT, Pyle Audio Inc., New York), chest strap electrocardiogram (ECG) sensor (MAX30003, San Jose, California), and blood pressure monitor (PHBPB20, Pyle Audio Inc., New York), respectively. The precision and accuracy are ±0.3°C and ±3  mmHg for IR thermometer and blood pressure monitor, respectively.

## 3 Results and Discussion

**Fig. 4:**
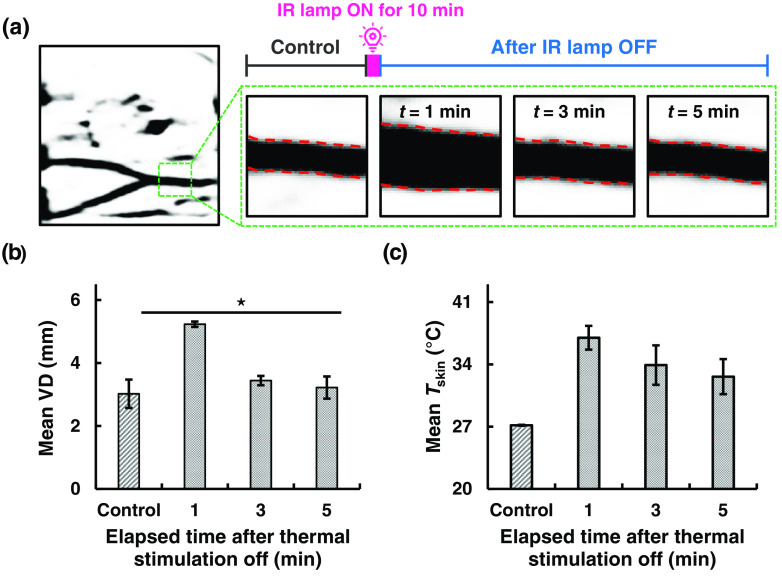
A repeatability study (n=2) of 2D vein dynamics of an individual at local heat exposure in study A condition. (a) A small region of the time sequence image is further cropped to magnify the vein dynamics due to local heat stimulation. The veins are outlined by red dashed lines in the time sequence images, where the control represents the vein image before heating and the change of the VD can be observed at t=1, 3, and 5 min after 10 min of local heat exposure. (b) Mean VD was measured at each time point from inset images in (a). A significant difference of VD can be seen from the bar chart. This significant difference was further justified by Student’s t-test (p<0.001). ⋆ indicates the significant difference (p≤0.001) between mean VDs measured before and after local heat exposure. (c) Mean $T_{\text{skin}}$ is plotted at t=1, 3, and 5 min after 10-min thermal stimulation is off and compared with the control.

An individual response on local heating is explained in Fig. 4. The dorsal surface of the hand was exposed to the noncontact radiation heat to observe the vein dynamics within the ROI. Figure 4(a) shows an image to be analyzed in the time sequence. The inset images were acquired at four different time points before and after local heat exposure. After the 10-min thermal stimulation was off, the vein images taken at 1, 3, and 5 min demonstrated the effect of thermal stress and relaxation for an individual. A subject participated in the experiment multiple times on separate days to test the repeatability of the VD dilation after thermal stress was applied. The mean VD from multiple experiments is plotted against elapsed time after the IR lamp is off in Fig. 4(b). After the thermal stimulation was off, it was clearly observed that VD was increased at 1 min compared with the control and then approached its original value after 5 min. To justify its significance, a Student’s t-test (p<0.001) was performed using IBM SPSS (v.25). We observed a similar trend for $T_{\text{skin}}$ with thermal stimulation as shown in Fig. 4(c). $T_{\text{skin}}$ did not return to its original value within 5 min after the thermal stimulation was off. The VD slowly decreased down to its original state within 5 min after the thermal stimulation was off. Thus, this information could be helpful for medical practitioners to try on intravenous procedures.

In this study, we demonstrated that a 10-min thermal stimulus using a noncontact IR radiant heater was safe and effective at inducing vasodilation in the dorsal surface of the hand of the participants. Our study also provides new and important information regarding the clinically relevant changes in $T_{\text{skin}}$ and vasodilation of the dorsal part of the hand induced by thermal stimulation. $T_{\text{skin}}$ was significantly higher at each time point after the thermal stimulus when compared with that before the stimulus. This result conforms with the findings of previous reports.^1^^,^^10^

Our results also indicate that a 10-min thermal stimulus for achieving $T_{\text{skin}}\approx 42{}^{\circ}C$ at the dorsal surface of the hand caused vasodilation to last at least for 4 min. This result is in line with the previous study that investigated the effect of thermal stimulation on cutaneous blood flow in the foot.^15^ While previous studies investigating the effect of thermal stimulation in the forearm measured VD before and after the thermal stimulus and compared the two time points, we measured VD for 5 min with an interval of 1 min after the thermal stimulus ends. Thus, we were able to show the time-dependent changes of vasodilation induced by the 10-min thermal stimulation.

**Fig. 5:**
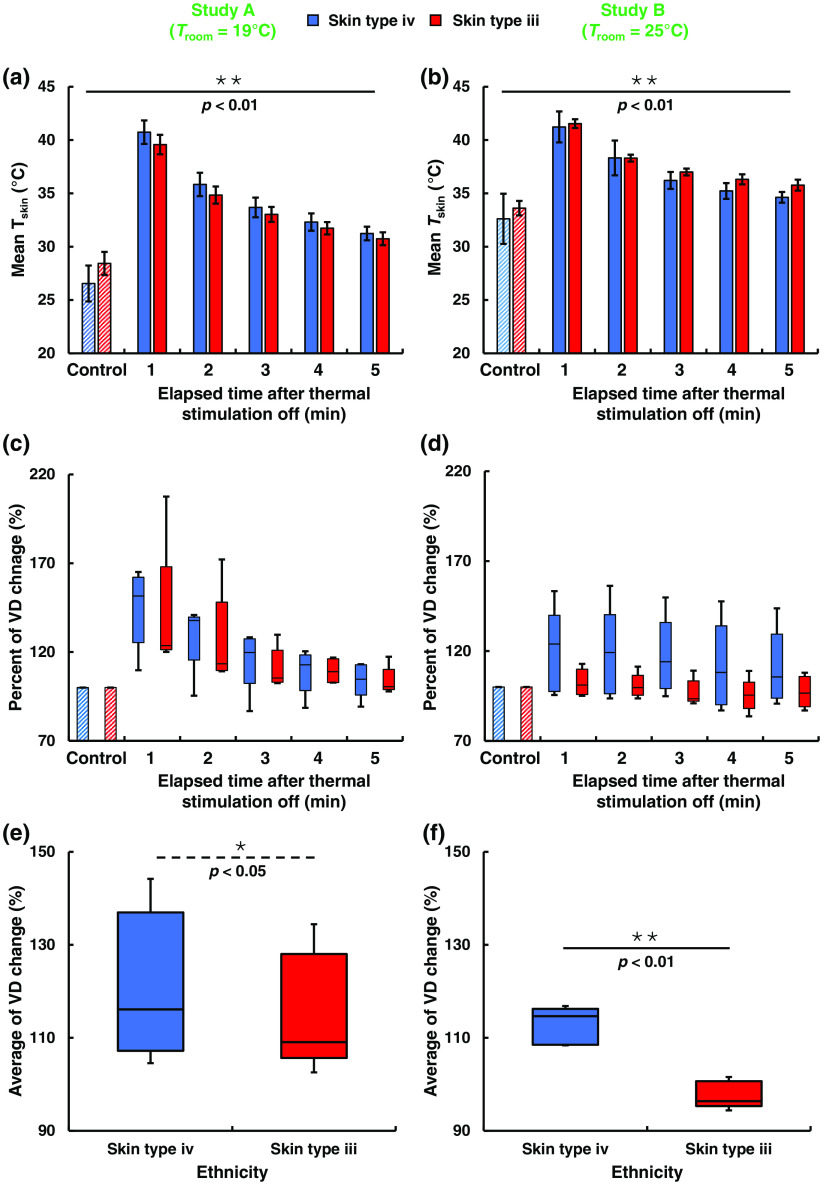
Before and after thermal stimulation effect on $T_{\text{skin}}$ and VD. (a) and (b) Values are shown as the average of the participants’ $T_{\text{skin}}$ at studies A and B (n=10). After the radiation heat exposure, there was a significant difference observed for $T_{\text{skin}}$ as shown in the bar chart. (c) and (d) Vein dynamics observed from its percent of dilation for different skin types. The vein was dilated after heat exposure to the dorsal hand surface. The standard deviation of VD was big as each individual has a different percentage of the dilation from their base diameter. However, the individual change of each person has a significant difference from their base value. (e) and (f) Time average of VD change shows a significant difference between two different races as confirmed by Student’s t-test. * and ** indicate the significant difference of p-value at p≤0.01 and p≤0.05, respectively.

Changes in blood flow in the skin during a response to a thermal stress are already well documented.^21^ However, the present study contributes further to the understanding of venous behavior during thermal stimulation. For example, our study utilized two different room temperatures. Moreover, Fig. 5 shows the changes in the skin and VD before and after the thermal stimulation for all the participants, including members of two races (Asian men and males from tropical region). The participants are grouped by their skin tones recommended by Fitzpatrick skin types. An Asian man and a male from tropical region are categorized as “skin type iii” and “skin type iv,” respectively. $T_{\text{skin}}$ decreased every minute after thermal stimulation was off in a similar manner for both values of $T_{\text{room}}$ and for both racial groups of participants, as shown in Figs. 5(a) and 5(b). A significant (p≤0.01) change in the temperature profile was observed for both values of $T_{\text{room}}$. Figures 5(c) and 5(d) show the averages of all participants’ VDs before and after the thermal stimulation. In study B, however, the VD did not change as much as study A for skin type iv participants and VD remained almost similar before and after thermal stimulation for skin type iii participants.

We found a significant (p≤0.001) difference between the $T_{\text{skin}}$ values before and after the noncontact heat stimulation. The VD after heat stimulation also was significantly (p≤0.01) different from the initial (“before heat stimulation”) value in study A, conformed by Student’s t-test. We measured the average VD before the thermal stimulation and used it as a reference (the 100% level), as shown in Fig. 5(c). One minute after the end of the thermal stimulation, the VD had changed from the initial value of 2.29±0.45  mm to 3.19±0.53  mm, and it then declined to 2.88±0.57  mm, 2.58±0.56  mm, 2.5±0.56  mm, and 2.37±0.46  mm at t=2, 3, 4, and 5 min, respectively. In study B, however, no significant difference was observed in the average vein dilation, as shown in Fig. 5(d). The base VD for study B was measured to be 3.08±0.45  mm before applying the thermal stimulation (considered as the 100% level) to compare with the values of VD after the thermal stimulation. We found that the VDs at 1 to 5 min after the thermal stimulation were 3.35±0.44  mm, 3.3±0.46  mm, 3.23±0.5  mm, 3.11±0.46  mm, and 3.14±0.46  mm, respectively. The reason for this difference may be because the initial VD and $T_{\text{skin}}$ were higher in study B than in study A. Thus, the VD evidently depends upon both $T_{\text{skin}}$ and $T_{\text{room}}$.

The control mean $T_{\text{skin}}$ of skin type iii men has a higher mean $T_{\text{skin}}$ over skin type iv in both studies groups A and B. This is well documented in several previous studies that Asian men have higher mean $T_{\text{skin}}$ over Tropical men.^22^ In our study, the mean $T_{\text{skin}}$ at control supports this finding. However, there is no notable change after thermal stimulation though the mean $T_{\text{skin}}$ was higher for the skin type iv men at study A after thermal stimulation and an opposite observation was found in study B condition. The mean $T_{\text{skin}}$ difference between these two races has no significant difference as confirmed by Student’s *t*-test.

In our study, two ethnic groups of people participated, and the response after the thermal stimulation differs in each group, as shown in Figs. 5(c) and 5(d). The number of participants was the same for both ethnic groups. Five skin type iii (Korean) males and five skin type iv (Indian and Bangladeshi) males participated in our study. We observed that the skin type iv men had more vein dilation after a thermal stimulation than skin type iii men in our study. In studies A and B, the significant (p≥0.03 and p≥0.001) difference was confirmed by a two tail Student’s t-test. This could happen due to the thermal tolerance of the individual’s race.^23^ Our result conforms with the suggestion by several studies that the skin type iv and skin type iii people have different thermal stress tolerances and their thermal responses are not identical even under the same environmental conditions.^24^

**Fig. 6:**
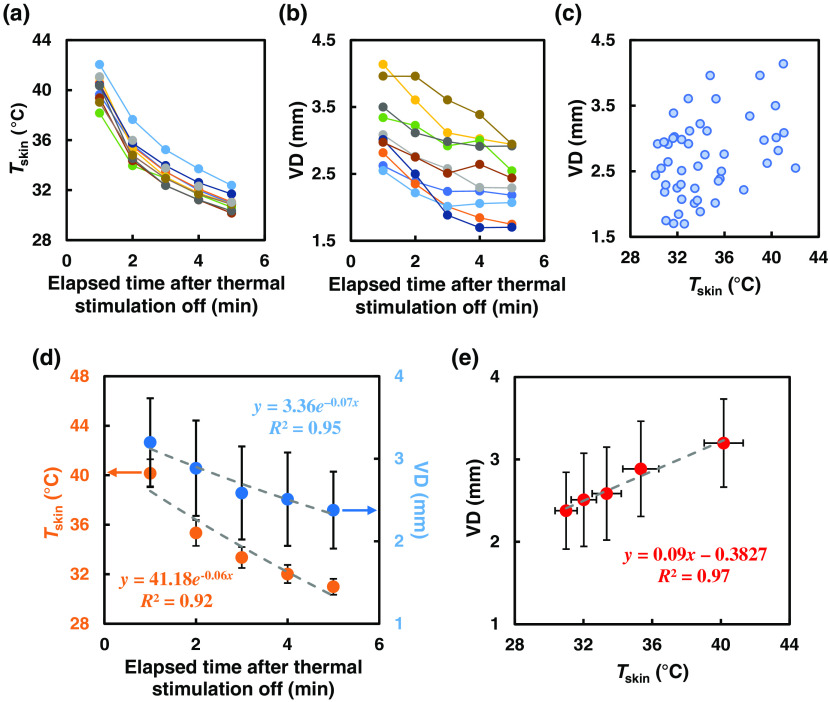
(a) and (b) Individual data points of $T_{\text{skin}}$ and VD for 5 min after thermal stimulation off. (c) A scatter plot that shows the VD change with change of $T_{\text{skin}}$ regardless of the vein size and race. (d) Mean $T_{\text{skin}}$ and VD exponentially decrease with time after removing the thermal stimulation in study A condition (n=10). (e) Linear correlation between the $T_{\text{skin}}$ and VD is shown, and the $R^{2}$ value indicates a good agreement between them.

The maximum VD was calculated from the maximum measured value divided by the baseline value [%DV = (maximum DV)/(base DV) ×100].^25^^,^^26^ We observed that study B, at a moderately higher $T_{\text{room}}$, did not produce a significant change in the VD in young healthy males after the thermal stimulation. At low $T_{\text{room}}$ (study A), we observed a relationship between the VD and $T_{\text{skin}}$ after the thermal stimulation. Data points are plotted in Figs. 6(a) and 6(b) for individual $T_{\text{skin}}$ and VD in response to elapsed time after thermal stimulation was off in study A condition. A scatter plot is shown in Fig. 6(c) for VD response regardless of $T_{\text{skin}}$ for all the races and the sizes of the vein. An exponential decrease was observed in both the VD and $T_{\text{skin}}$ during 5 min after the thermal stimulation, with $R^{2}>0.9$, as shown in Fig. 6(d). We analyzed the data further to determine the correlation between the $T_{\text{skin}}$ and VD and found $R^{2}>0.9$ as shown in Fig. 6(e), which confirms the fairly good correlation ($R^{2}=0.97$) between the $T_{\text{skin}}$ and VD.

**Fig. 7:**
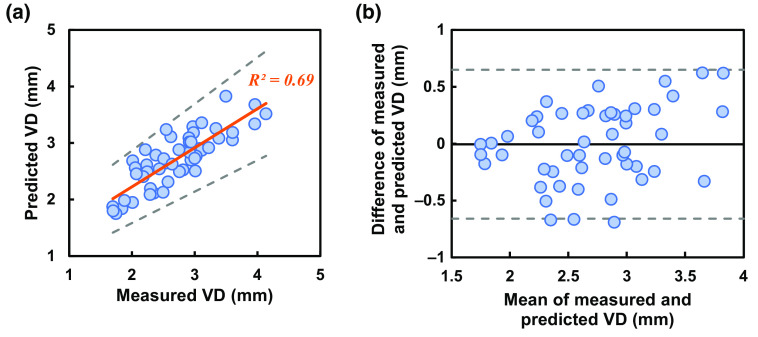
(a) A multilinear-regression model was made and validated with scattered plot as shown where $R^{2}=0.69$. (b) Bland–Altman plot analysis of the prediction model shows ±0.6  mm prediction variance form the measured value.

The observation ended after 5 min of thermal stimulation when the vein returned to its initial size. Our finding shows that a regression model can predict the final VD before applying the thermal stimulation in study A condition. As a result, we made a multiple linear-regression model as shown in Eq. (1)

$${\mathrm{VD}}_{\mathrm{pre}}=-17.9+0.28(T_{\mathrm{ini}\_\mathrm{skin}})+0.93({\mathrm{VD}}_{\mathrm{ini}})+0.37(T_{\mathrm{fin}\_\mathrm{skin}}),$$ (1)

where ${\mathrm{VD}}_{\text{pre}}=\mathrm{predicted}$ VD, $T_{\mathrm{fin}\_\mathrm{skin}}=\text{initial skin temperature}$, ${\mathrm{VD}}_{\text{ini}}=\mathrm{initial}$ VD, and $T_{\mathrm{fin}\_\mathrm{skin}}=\text{final skin temperature}$. Equation (1) can be used to predict VD after thermal stimulation. A scatter plot is shown in Fig. 7(a) for predicted versus measured mean VD after thermal stimulation. The $R^{2}$ value is 0.69 between the predicted and measured mean VD. A Bland–Altman scatter plot is drawn by measuring the mean of measured and predicted values against the difference of the measured and predicted values. The limit of agreement from the base is ±0.6  mm as observed in Fig. 7(b).

There are several limitations in the present pilot study. First, we only recruited young and healthy volunteers. This is particularly relevant because compared with young individuals, older individuals have relatively fragile veins.^27^^,^^28^ Moreover, no obese person participated in our study, so further studies are needed to assess the effect of body-mass index (BMI).

There was no significant difference in other physiological signals as listed in Table 2. The ${\mathrm{BP}}_{\text{sys}}$ exhibited no significant change after the thermal stimulation in either studies A or B. In addition, $T_{\text{tym}}$ and HR did not display any significant change after the local thermal stimulation. However, a small change was observed between the two initial study conditions; the systolic BP was observed less at study B than study A with a significant difference (p<0.01). A similar case of decreasing systolic BP at higher ambient temperature was observed in another study.^29^ However, $T_{\text{tym}}$ increased at higher $T_{\text{room}}$ with a significant difference (p<0.001) as shown in Table 2. In addition, we performed our experiment only with male subjects, so our study provides no information about the female response. More experiments should be conducted in the future to observe the response of female as well as male participants. In our experiment, not all the participants responded equally to the thermal stimulation. An increase in the mean VD because of an increase in local skin temperature is not the result of chance alone, but rather it is statistically significant, as stated by Irfan et al.^30^ Different artery and vein sizes may vary tremendously in response to body posture, stress, and external environment.^31^^,^^32^ There also may be other effects on vein dynamics that we have not discussed here as we have restricted our study to local thermal stimulation in a controlled environment, keeping in mind the goal of helping health workers to improve VP and VC on the patient.

**Table 2:** Vital signs measured before and after applying thermal stimulation.

Vital sign	Study A		Study B		p-value
	Before	After	Before	After	A and B
Systolic BP (mmHg)	134±18	131±16	124±13	125±12	<0.01
Body temperature (°C)	35.7±0.2	35.8±0.2	36.3±0.1	36.4	<0.001
Pulse (BPM)	70±15	67±9	72±10	77±19	—

Note: All data were presented mean ± SD (standard deviation). n=8; BP = blood pressure, BPM = beats per min, p = significance of Student’s t-test

## 4 Conclusions

Our results suggest that local heat application helps to increase the vessel diameter. It can enhance the intravenous catheter insertion, decrease the procedure time, and improve the quality of care. Based on our findings, a noncontact IR radiant heater can be used alternatively to a contact-based heater. $T_{\text{skin}}$ and VD have a proportional change after heat exposure. The veins remain dilated a few more minutes after the local heat is removed from the site. An empirical prediction model is deduced in a controlled condition for predicting final VD. Knowing the initial $T_{\text{skin}}$, VD and desired final $T_{\text{skin}}$ can potentially help find the desired VD. However, skin type iv men have higher vein dilation response to thermal stimulation than skin type iii men. This finding can be further extended to develop a real-time image-guided medical device by integrating with a radiant heater to assist medical practitioner for a high success rate of VP or VC.
